# Supplementary material for: Explosive percolation yields highly-conductive polymer nanocomposites
Source: Nat Commun. 2022 Nov 11;13:6872. doi: 10.1038/s41467-022-34631-9 (PMC9652282; doi:10.1038/s41467-022-34631-9)
Supplement: Supplementary file 1 — Supplementary Information [file 41467_2022_34631_MOESM1_ESM.pdf]

Explosive percolation yields highly-conductive polymer nanocomposites

Manuela Meloni[1], Matthew J. Large[1*], José Miguel González Domínguez[2], Sandra Victor-Román[2], Giuseppe Fratta[1], Emin Istif[2], Oliver Tomes[1], Jonathan P. Salvage[3], Christopher P. Ewels[4], Mario Pelaez-Fernandez[5], Raul Arenal[5,6,7], Ana Benito[2], Wolfgang K. Maser[2], Alice A. K. King[1], Pulickel Ajayan[8], Sean P. Ogilvie[1*] and Alan B. Dalton[1*]

Supplementary information

Table of references for main text Figure 1H and 1I

Supplementary Table 1 gives references for the data plotted in Figure 1H of the main text (Comparison of the maximum reported conductivity (and corresponding filler content) for 25 composites from the literature, categorised by filler material).

Supplementary Table 1: references for the data plotted in Figure 1H of the main text.

Matrix	Filler	Loading level /wt%	Conductivity /S/m	Reference
PMMA-co-BA	Carbon black	24	10	[1]
PP	Carbon black	10	1.5	[2]
PVAc	Carbon black	40	33.8	[3]
Epoxy	Cu	8.96	3.06	[4]
PVAc	SWCNT	4	25	[5]
PS	MWCNT	5.5	1	[6]
BA-MMA-AAEM	SWCNT	1	10	[7]
PP	MWCNT	1	1.5	[2]
PVDF	MWCNT-Ag	8.6	571	[8]
PC	Graphene	4.4	51.2	[9]
PS	Graphene	2	15	[10]
PP	Graphene	3.5	1.5	[2]
PS	Graphene	2.4	25.2	[11]
PS-EVA	Graphene	9.6	1024	[12]
PA6	Graphene	4.9	60	[13]
Cellulose	Graphene	10	71.8	[14]
PMMA-co-BA	Graphene	22	700	[15]
PMMA	Graphene	4	1	[16]
PP	Graphite	10	0.1	[2]
PS	rGO	2	12	[17]
PMMA	rGO	5.4	64	[18]
PP	rGO	2	0.4	[17]
PS	rGO	8	20.5	[19]
PVP	rGO	0.9	0.23	[20]
PTFE	rGO	2	1.4	[21]

Supplementary Table 2 gives reference for the data plotted in Figure 1I of the main text (Maximum reported conductivity of polymer composites of rGO and pristine graphene from the literature against reported percolation threshold). Some percolation threshold filler contents originally reported in volume percent have been converted to weight percent for consistency.

Supplementary Table 2: references for the data plotted in Figure 1I of the main text

Matrix	Filler	Percolation threshold /wt%	Ultimate conductivity /S/m	Reference
Reduced graphene oxide (rGO) materials				
ABS	GO	0.2925	0.1	[22]
PA6	TRGO	7.5	0.0071	[23]
PA6	rGO	0.9225	0.028	[24]
PA12	TRGO	1	0.0001	[25]
PA12	TRGO	2.5	0.089	[26]
PC	TRGO	2.5	0.1	[23]
PC	rGO	0.2	0.1	[27]
LLDPE	TRGO	1.125	0.0001	[28]
UHMWPE	rGO	0.063	5	[29]
PLA	TRGO	1.125	0.01	[30]
PMMA	rGO	0.5625	0.01	[31]
PP	TRGO	5	0.0001	[32]
PS	rGO	0.45	0.05	[33]
PU	rGO	0.1755	0.001	[34]
Epoxy	CRGO aerogel	0.25	20	[35]
Epoxy	TRGO	1	0.000002	[36]
Epoxy	rGO	0.27	1	[37]
NR	rGO	0.5175	1	[38]
NR	rGO	0.4725	0.23	[39]
Pristine graphene materials				
PS	Graphene	0.18	25.2	[11]
PS-EVA	Graphene	0.3	1024	[12]
PMMA	Graphene	0.6	1	[16]
PMMA-co-BA	Graphene	0.8	700	[15]
PP	Graphene	1.2	1.5	[2]
PA6	Graphene	0.064	60	[13]
Cellulose	Graphene	0.3	71.8	[14]
PS	Graphene	0.7425	3.5	[40]
PS-PLA	Graphene	0.16875	3	[40]
NR	Graphene	1.395	0.03	[41]
PLA	Graphene	0.009	0.1	[30]
PMMA-co-BA	Graphene	0.225	217	[31]
PS	Graphene	0.225	13.8	[42]
PS	Graphene	0.2025	25.2	[11]
sPS	Graphene	1.035	470	[43]
PS	Graphene	0.1215	46	[44]
PVC	Graphene	0.225	5.8	[45]
Epoxy	Graphene	1.125	0.01	[46]
Epoxy	Graphene	1.17	0.05	[47]

Comparison of explosive and isotropic percolating systems

Supplementary Figure 1**A** modifies a plot from [48] comparing the scaling of systems exhibiting explosive percolation (red) to those exhibiting isotropic percolation (black) in schematic form.

As a close experimental analogue to the theoretical cases, Supplementary Figure 1**B** plots normalised conductivity data from Figure 1**E** of the main text ("*In-situ* rGO") against conductivity data from our prior work on liquid-phase exfoliated graphene, which was performed using the same matrix polymer [49]. The normalisation has been performed by extrapolating the fitted percolation curves to 100% filler loading and dividing the experimental data by that value (i.e. the conductivity of the conductive network) to produce curves representing the fraction of the maximum system conductivity as a function of filler loading.

As can be seen in Supplementary Figure 1**B**, the experimental data follow both the trends and display the comparative properties expected for systems which obey isotropic percolation (LPE graphene) and explosive percolation (*in-situ* rGO, this work), both in terms of their relative percolation thresholds (0.15wt% *cf.* 0.40wt%) and relative critical scaling exponents (3.8 *cf.* 0.6).

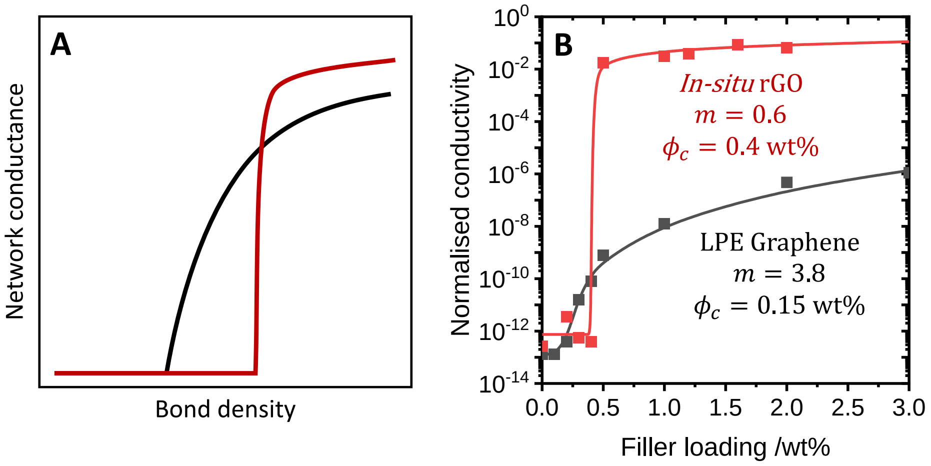

Supplementary Figure 1: **A**. Schematic comparison of explosive (red) and isotropic (black) percolative systems, modified from the literature [48]. **B**. Plot of conductivity normalised to the predicted 100wt% loading for in-situ rGO (this work) and LPE graphene [49]. Both data sets use the same polymer and filler particles of comparable aspect ratio.

Differential Scanning Calorimetry data

Supplementary Figure 2 shows DSC data covering the endothermic melting peak which corresponds to the exothermic melting peak presenting the main text (Figure 2**F**). As can be seen, the melting peak does not undergo any significant changes as a consequence of the addition of GO flakes to the system, unlike the crystallisation peak which exhibits an additional higher-temperature feature emerging after reduction of the GO to rGO *in-situ*.

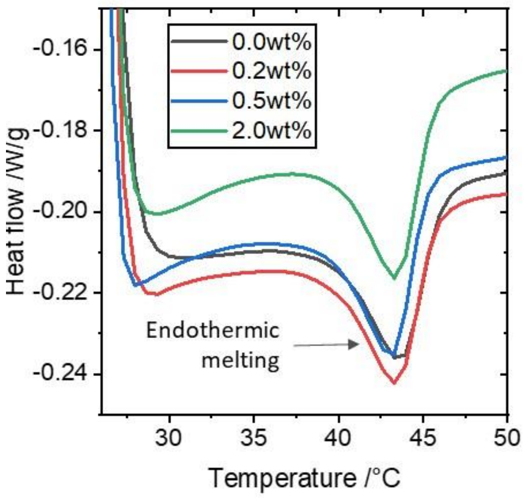

Supplementary Figure 2: DSC data showing the endothermic melting feature associated with the exothermic crystallisation feature in the main text Figure 2**F**.

Graphene oxide filler characterisation data

Supplementary Figure 3 shows a particle size histogram of the GO used in this work, with a typical AFM image inset. Supplementary Figure 4 shows the reference C1s and O1s spectra for the GO used in this work.

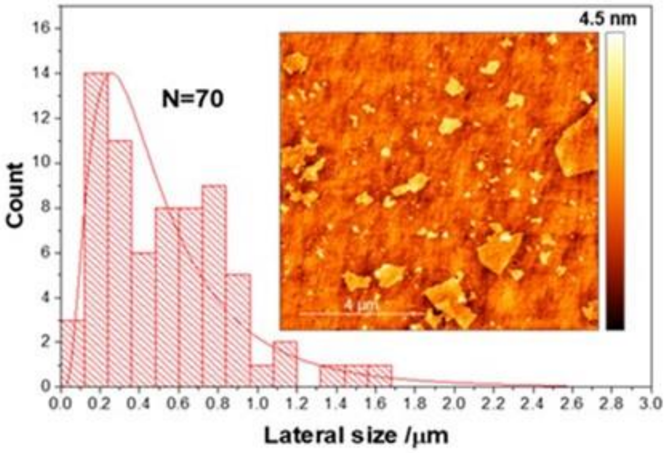

Supplementary Figure 3: Particle size distribution of GO and AFM image in topography of GO flakes.

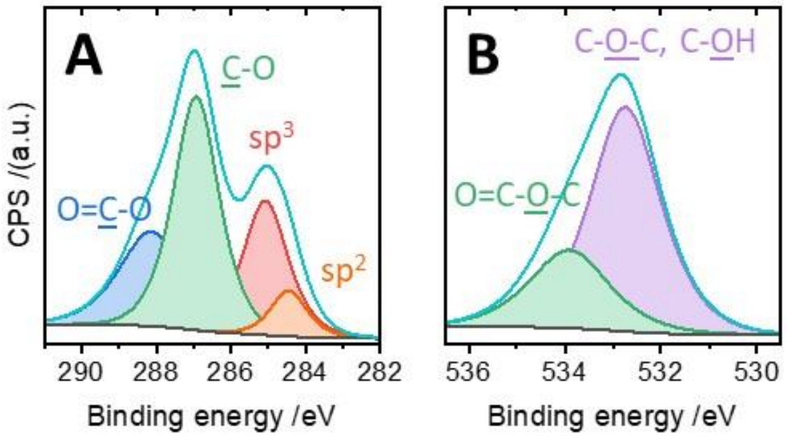

Supplementary Figure 4. High resolution C1*s* spectrum a) (with peaks labelled as in the main text, Figure 4A); and O1*s* spectrum b) (with peaks as labelled in main text, Figure 4E) of the as-used GO.

References

[1] J. C. Grunlan, Y. Ma, M. A. Grunlan, W. W. Gerberich, and L. F. Francis, 'Monodisperse latex with variable glass transition temperature and particle size for use as matrix starting material for conductive polymer composites', *Polymer*, vol. 42, no. 16, pp. 6913–6921, Jul. 2001, doi: 10.1016/S0032-3861(01)00158-6.

[2] M. Ghislandi, E. Tkalya, B. Marinho, C. E. Koning, and G. de With, 'Electrical conductivities of carbon powder nanofillers and their latex-based polymer composites', *Compos. Part Appl. Sci. Manuf.*, vol. 53, pp. 145–151, Oct. 2013, doi: 10.1016/j.compositesa.2013.06.008.

[3] J. C. Grunlan, W. W. Gerberich, and L. F. Francis, 'Electrical and mechanical behavior of carbon black-filled poly(vinyl acetate) latex-based composites', *Polym. Eng. Sci.*, vol. 41, no. 11, pp. 1947–1962, Nov. 2001, doi: 10.1002/pen.10891.

[4] N. H. Mohd Hirmizi, M. Abu Bakar, W. L. Tan, N. H. H. Abu Bakar, J. Ismail, and C. H. See, 'Electrical and Thermal Behavior of Copper-Epoxy Nanocomposites Prepared via Aqueous to Organic Phase Transfer Technique', *J. Nanomater.*, vol. 2012, pp. 1–11, 2012, doi: 10.1155/2012/219073.

[5] J. C. Grunlan, A. R. Mehrabi, M. V. Bannon, and J. L. Bahr, 'Water-Based Single-Walled-Nanotube-Filled Polymer Composite with an Exceptionally Low Percolation Threshold', *Adv. Mater.*, vol. 16, no. 2, pp. 150–153, Jan. 2004, doi: 10.1002/adma.200305409.

[6] J. Yu, K. Lu, E. Sourty, N. Grossiord, C. E. Koning, and J. Loos, 'Characterization of conductive multiwall carbon nanotube/polystyrene composites prepared by latex technology', *Carbon*, vol. 45, no. 15, pp. 2897–2903, Dec. 2007, doi: 10.1016/j.carbon.2007.10.005.

[7] I. Jurewicz, P. Worajittiphon, A. A. K. King, P. J. Sellin, J. L. Keddie, and A. B. Dalton, 'Locking Carbon Nanotubes in Confined Lattice Geometries − A Route to Low Percolation in Conducting Composites', *J. Phys. Chem. B*, vol. 115, no. 20, pp. 6395–6400, May 2011, doi: 10.1021/jp111998p.

[8] K.-Y. Chun *et al.*, 'Highly conductive, printable and stretchable composite films of carbon nanotubes and silver', *Nat. Nanotechnol.*, vol. 5, no. 12, pp. 853–857, Dec. 2010, doi: 10.1038/nnano.2010.232.

[9] M. Yoonessi and J. R. Gaier, 'Highly Conductive Multifunctional Graphene Polycarbonate Nanocomposites', *ACS Nano*, vol. 4, no. 12, pp. 7211–7220, Dec. 2010, doi: 10.1021/nn1019626.

[10] E. Tkalya, M. Ghislandi, A. Alekseev, C. Koning, and J. Loos, 'Latex-based concept for the preparation of graphene-based polymer nanocomposites', *J. Mater. Chem.*, vol. 20, no. 15, p. 3035, 2010, doi: 10.1039/b922604d.

[11] P. Zhao *et al.*, 'Electrically conductive graphene-filled polymer composites with well organized three-dimensional microstructure', *Mater. Lett.*, vol. 121, pp. 74–77, Apr. 2014, doi: 10.1016/j.matlet.2014.01.100.

[12] C. Wu *et al.*, 'Highly Conductive Nanocomposites with Three-Dimensional, Compactly Interconnected Graphene Networks via a Self-Assembly Process', *Adv. Funct. Mater.*, vol. 23, no. 4, pp. 506–513, Jan. 2013, doi: 10.1002/adfm.201201231.

[13] P. Wang, H. Chong, J. Zhang, and H. Lu, 'Constructing 3D Graphene Networks in Polymer Composites for Significantly Improved Electrical and Mechanical Properties', *ACS Appl. Mater. Interfaces*, vol. 9, no. 26, pp. 22006–22017, Jul. 2017, doi: 10.1021/acsami.7b07328.

[14] N. D. Luong *et al.*, 'Graphene/cellulose nanocomposite paper with high electrical and mechanical performances', *J. Mater. Chem.*, vol. 21, no. 36, p. 13991, 2011, doi: 10.1039/c1jm12134k.

[15] A. Noël, J. Faucheu, J.-M. Chenal, J.-P. Viricelle, and E. Bourgeat-Lami, 'Electrical and mechanical percolation in graphene-latex nanocomposites', *Polymer*, vol. 55, no. 20, pp. 5140–5145, Sep. 2014, doi: 10.1016/j.polymer.2014.08.025.

[16] G. Chen, W. Weng, D. Wu, and C. Wu, ‘PMMA/graphite nanosheets composite and its conducting properties’, *Eur. Polym. J.*, vol. 39, no. 12, pp. 2329–2335, Dec. 2003, doi: 10.1016/j.eurpolymj.2003.08.005.
[17] Y. V. Syurik *et al.*, ‘Graphene Network Organisation in Conductive Polymer Composites’, *Macromol. Chem. Phys.*, vol. 213, no. 12, pp. 1251–1258, Jun. 2012, doi: 10.1002/macp.201200116.
[18] V. H. Pham, T. T. Dang, S. H. Hur, E. J. Kim, and J. S. Chung, ‘Highly Conductive Poly(methyl methacrylate) (PMMA)-Reduced Graphene Oxide Composite Prepared by Self-Assembly of PMMA Latex and Graphene Oxide through Electrostatic Interaction’, *ACS Appl. Mater. Interfaces*, vol. 4, no. 5, pp. 2630–2636, May 2012, doi: 10.1021/am300297j.
[19] G. Long *et al.*, ‘Resolving the dilemma of gaining conductivity but losing environmental friendliness in producing polystyrene/graphene composites via optimizing the matrix-filler structure’, *Green Chem.*, vol. 15, no. 3, p. 821, 2013, doi: 10.1039/c3gc37042a.
[20] A. Arzac, G. P. Leal, R. Fajgar, and R. Tomovska, ‘Comparison of the Emulsion Mixing and In Situ Polymerization Techniques for Synthesis of Water-Borne Reduced Graphene Oxide/Polymer Composites: Advantages and Drawbacks’, *Part. Part. Syst. Charact.*, vol. 31, no. 1, pp. 143–151, Jan. 2014, doi: 10.1002/ppsc.201300286.
[21] H. Jiang, L. Chen, S. Chai, X. Yao, F. Chen, and Q. Fu, ‘Facile fabrication of poly (tetrafluoroethylene)/graphene nanocomposite via electrostatic self-assembly approach’, *Compos. Sci. Technol.*, vol. 103, pp. 28–35, Oct. 2014, doi: 10.1016/j.compscitech.2014.08.007.
[22] C. Gao *et al.*, ‘Graphene Networks with Low Percolation Threshold in ABS Nanocomposites: Selective Localization and Electrical and Rheological Properties’, *ACS Appl. Mater. Interfaces*, vol. 6, no. 15, pp. 12252–12260, Aug. 2014, doi: 10.1021/am501843s.
[23] P. Steurer, R. Wissert, R. Thomann, and R. Mülhaupt, ‘Functionalized Graphenes and Thermoplastic Nanocomposites Based upon Expanded Graphite Oxide’, *Macromol. Rapid Commun.*, vol. 30, no. 4–5, pp. 316–327, Feb. 2009, doi: 10.1002/marc.200800754.
[24] B. J. Rashmi, K. Prashantha, M.-F. Lacrampe, and P. Krawczak, ‘Scalable Production of Multifunctional Bio-Based Polyamide 11/Graphene Nanocomposites by Melt Extrusion Processes Via Masterbatch Approach’, *Adv. Polym. Technol.*, vol. 37, no. 4, pp. 1067–1075, Jun. 2018, doi: 10.1002/adv.21757.
[25] M. Beckert, F. J. Tölle, B. Bruchmann, and R. Mülhaupt, ‘Nitrogen-Doped Multilayer Graphene as Functional Filler for Carbon/Polyamide 12 Nanocomposites: Nitrogen-Doped Multilayer Graphene as Functional Filler’, *Macromol. Mater. Eng.*, vol. 300, no. 8, pp. 785–792, Aug. 2015, doi: 10.1002/mame.201500020.
[26] D. Hofmann, M. Keinath, R. Thomann, and R. Mülhaupt, ‘Thermoplastic Carbon/Polyamide 12 Composites Containing Functionalized Graphene, Expanded Graphite, and Carbon Nanofillers: Thermoplastic Carbon/Polyamide 12 Composites Containing Functionalized Graphene …’, *Macromol. Mater. Eng.*, vol. 299, no. 11, pp. 1329–1342, Nov. 2014, doi: 10.1002/mame.201400066.
[27] C. Xu *et al.*, ‘Can in situ thermal reduction be a green and efficient way in the fabrication of electrically conductive polymer/reduced graphene oxide nanocomposites?’, *Compos. Part Appl. Sci. Manuf.*, vol. 53, pp. 24–33, Oct. 2013, doi: 10.1016/j.compositesa.2013.06.007.
[28] A. A. Vasileiou, M. Kontopoulou, and A. Docoslis, ‘A Noncovalent Compatibilization Approach to Improve the Filler Dispersion and Properties of Polyethylene/Graphene Composites’, *ACS Appl. Mater. Interfaces*, vol. 6, no. 3, pp. 1916–1925, Feb. 2014, doi: 10.1021/am404979g.
[29] H. Hu, G. Zhang, L. Xiao, H. Wang, Q. Zhang, and Z. Zhao, ‘Preparation and electrical conductivity of graphene/ultrahigh molecular weight polyethylene composites with a segregated structure’, *Carbon*, vol. 50, no. 12, pp. 4596–4599, Oct. 2012, doi: 10.1016/j.carbon.2012.05.045.
[30] M. Sabzi, L. Jiang, F. Liu, I. Ghasemi, and M. Atai, ‘Graphene nanoplatelets as poly(lactic acid) modifier: linear rheological behavior and electrical conductivity’, *J. Mater. Chem. A*, vol. 1, no. 28, p. 8253, 2013, doi: 10.1039/c3ta11021d.

[31] A. Noël, J. Faucheu, M. Rieu, J.-P. Viricelle, and E. Bourgeat-Lami, ‘Tunable architecture for flexible and highly conductive graphene–polymer composites’, *Compos. Sci. Technol.*, vol. 95, pp. 82–88, May 2014, doi: 10.1016/j.compscitech.2014.02.013.
[32] D. Hofmann, K.-A. Wartig, R. Thomann, B. Dittrich, B. Schartel, and R. Mülhaupt, ‘Functionalized Graphene and Carbon Materials as Additives for Melt-Extruded Flame Retardant Polypropylene: Functionalized Graphene and Carbon Materials as Additives …’, *Macromol. Mater. Eng.*, vol. 298, no. 12, pp. 1322–1334, Dec. 2013, doi: 10.1002/mame.201200433.
[33] W. Fan, C. Zhang, W. W. Tjiu, and T. Liu, ‘Fabrication of electrically conductive graphene/polystyrene composites via a combination of latex and layer-by-layer assembly approaches’, *J. Mater. Res.*, vol. 28, no. 4, pp. 611–619, Feb. 2013, doi: 10.1557/jmr.2012.437.
[34] N. Yousefi, M. M. Gudarzi, Q. Zheng, S. H. Aboutalebi, F. Sharif, and J.-K. Kim, ‘Self-alignment and high electrical conductivity of ultralarge graphene oxide–polyurethane nanocomposites’, *J. Mater. Chem.*, vol. 22, no. 25, p. 12709, 2012, doi: 10.1039/c2jm30590a.
[35] Z. Wang *et al.*, ‘Graphene Aerogel/Epoxy Composites with Exceptional Anisotropic Structure and Properties’, *ACS Appl. Mater. Interfaces*, vol. 7, no. 9, pp. 5538–5549, Mar. 2015, doi: 10.1021/acsami.5b00146.
[36] K. Tschoppe, F. Beckert, M. Beckert, and R. Mülhaupt, ‘Thermally Reduced Graphite Oxide and Mechanochemically Functionalized Graphene as Functional Fillers for Epoxy Nanocomposites: Thermally Reduced Graphite Oxide and Mechanochemically …’, *Macromol. Mater. Eng.*, vol. 300, no. 2, pp. 140–152, Feb. 2015, doi: 10.1002/mame.201400245.
[37] N. Yousefi *et al.*, ‘Highly Aligned Graphene/Polymer Nanocomposites with Excellent Dielectric Properties for High-Performance Electromagnetic Interference Shielding’, *Adv. Mater.*, vol. 26, no. 31, pp. 5480–5487, Aug. 2014, doi: 10.1002/adma.201305293.
[38] C. He *et al.*, ‘Graphene networks and their influence on free-volume properties of graphene–epoxidized natural rubber composites with a segregated structure: rheological and positron annihilation studies’, *Phys. Chem. Chem. Phys.*, vol. 17, no. 18, pp. 12175–12184, 2015, doi: 10.1039/C5CP00465A.
[39] B. Dong, S. Wu, L. Zhang, and Y. Wu, ‘High Performance Natural Rubber Composites with Well-Organized Interconnected Graphene Networks for Strain-Sensing Application’, *Ind. Eng. Chem. Res.*, vol. 55, no. 17, pp. 4919–4929, May 2016, doi: 10.1021/acs.iecr.6b00214.
[40] X.-Y. Qi *et al.*, ‘Enhanced Electrical Conductivity in Polystyrene Nanocomposites at Ultra-Low Graphene Content’, *ACS Appl. Mater. Interfaces*, vol. 3, no. 8, pp. 3130–3133, Aug. 2011, doi: 10.1021/am200628c.
[41] Y. Zhan, M. Lavorgna, G. Buonocore, and H. Xia, ‘Enhancing electrical conductivity of rubber composites by constructing interconnected network of self-assembled graphene with latex mixing’, *J. Mater. Chem.*, vol. 22, no. 21, p. 10464, 2012, doi: 10.1039/c2jm31293j.
[42] N. Liu, F. Luo, H. Wu, Y. Liu, C. Zhang, and J. Chen, ‘One-Step Ionic-Liquid-Assisted Electrochemical Synthesis of Ionic-Liquid-Functionalized Graphene Sheets Directly from Graphite: Ionic-Liquid-Assisted Electrochemical Synthesis of Graphene’, *Adv. Funct. Mater.*, vol. 18, no. 10, pp. 1518–1525, May 2008, doi: 10.1002/adfm.200700797.
[43] Y.-C. Chiu, C.-L. Huang, and C. Wang, ‘Rheological and conductivity percolations of syndiotactic polystyrene composites filled with graphene nanosheets and carbon nanotubes: A comparative study’, *Compos. Sci. Technol.*, vol. 134, pp. 153–160, Oct. 2016, doi: 10.1016/j.compscitech.2016.08.016.
[44] Z. Tu *et al.*, ‘A facile approach for preparation of polystyrene/graphene nanocomposites with ultra-low percolation threshold through an electrostatic assembly process’, *Compos. Sci. Technol.*, vol. 134, pp. 49–56, Oct. 2016, doi: 10.1016/j.compscitech.2016.08.003.
[45] S. Vadukumpully, J. Paul, N. Mahanta, and S. Valiyaveettil, ‘Flexible conductive graphene/poly(vinyl chloride) composite thin films with high mechanical strength and thermal stability’, *Carbon*, vol. 49, no. 1, pp. 198–205, Jan. 2011, doi: 10.1016/j.carbon.2010.09.004.

[46] M. Monti, M. Rallini, D. Puglia, L. Peponi, L. Torre, and J. M. Kenny, 'Morphology and electrical properties of graphene–epoxy nanocomposites obtained by different solvent assisted processing methods', *Compos. Part Appl. Sci. Manuf.*, vol. 46, pp. 166–172, Mar. 2013, doi: 10.1016/j.compositesa.2012.11.005.
[47] Y. Li, H. Zhang, H. Porwal, Z. Huang, E. Bilotti, and T. Peijs, 'Mechanical, electrical and thermal properties of in-situ exfoliated graphene/epoxy nanocomposites', *Compos. Part Appl. Sci. Manuf.*, vol. 95, pp. 229–236, Apr. 2017, doi: 10.1016/j.compositesa.2017.01.007.
[48] R. M. D'Souza and J. Nagler, 'Anomalous critical and supercritical phenomena in explosive percolation', *Nat. Phys.*, vol. 11, no. 7, Art. no. 7, Jul. 2015, doi: 10.1038/nphys3378.
[49] I. Jurewicz *et al.*, 'Mechanochromic and thermochromic sensors based on graphene infused polymer opals', *Adv. Funct. Mater.*, pp. 1–38, Apr. 2020, doi: 10.1002/adfm.202002473.
